# Brain Macrophages in Simian Immunodeficiency Virus-Infected, Antiretroviral-Suppressed Macaques: a Functional Latent Reservoir

**DOI:** 10.1128/mBio.01186-17

**Published:** 2017-08-15

**Authors:** Claudia R. Avalos, Celina M. Abreu, Suzanne E. Queen, Ming Li, Sarah Price, Erin N. Shirk, Elizabeth L. Engle, Ellen Forsyth, Brandon T. Bullock, Feilim Mac Gabhann, Stephen W. Wietgrefe, Ashley T. Haase, M. Christine Zink, Joseph L. Mankowski, Janice E. Clements, Lucio Gama

**Affiliations:** a Department of Molecular and Comparative Pathobiology, Johns Hopkins School of Medicine, Baltimore, Maryland, USA; b Department of Pathology, Johns Hopkins School of Medicine, Baltimore, Maryland, USA; c Department of Neurology, Johns Hopkins School of Medicine, Baltimore, Maryland, USA; d Department of Microbiology and Immunology, University of Minnesota Medical School, Minneapolis, Minnesota, USA; e Department of Biomedical Engineering and Institute for Computational Medicine, Johns Hopkins University, Baltimore, Maryland, USA; Johns Hopkins Bloomberg School of Public Health

**Keywords:** brain, human immunodeficiency virus, latency, macrophages, simian immunodeficiency virus

## Abstract

A human immunodeficiency virus (HIV) infection cure requires an understanding of the cellular and anatomical sites harboring virus that contribute to viral rebound upon treatment interruption. Despite antiretroviral therapy (ART), HIV-associated neurocognitive disorders (HAND) are reported in HIV-infected individuals on ART. Biomarkers for macrophage activation and neuronal damage in cerebrospinal fluid (CSF) of HIV-infected individuals demonstrate continued effects of HIV in brain and suggest that the central nervous system (CNS) may serve as a viral reservoir. Using a simian immunodeficiency virus (SIV)/macaque model for HIV encephalitis and AIDS, we evaluated whether infected cells persist in brain despite ART. Eight SIV-infected pig-tailed macaques were virally suppressed with ART, and plasma and CSF viremia levels were analyzed longitudinally. To assess whether virus persisted in brain macrophages (BrMΦ) in these macaques, we used a macrophage quantitative viral outgrowth assay (MΦ-QVOA), PCR, and *in situ* hybridization (ISH) to measure the frequency of infected cells and the levels of viral RNA and DNA in brain. Viral RNA in brain tissue of suppressed macaques was undetectable, although viral DNA was detected in all animals. The MΦ-QVOA demonstrated that the majority of suppressed animals contained latently infected BrMΦ. We also showed that virus produced in the MΦ-QVOAs was replication competent, suggesting that latently infected BrMΦ are capable of reestablishing productive infection upon treatment interruption. This report provides the first confirmation of the presence of replication-competent SIV in BrMΦ of ART-suppressed macaques and suggests that the highly debated issue of viral latency in macrophages, at least in brain, has been addressed in SIV-infected macaques treated with ART.

## IMPORTANCE

Resting CD4^+^ T cells are currently the only cells that fit the definition of a latent reservoir. However, recent evidence suggests that HIV/SIV-infected macrophages persist despite ART. Markers of macrophage activation and neuronal damage are observed in the CSF of HIV-infected individuals and of SIV-infected macaques on suppressive ART regimens, suggesting that the CNS has continued virus infection and latent infection. A controversy exists as to whether brain macrophages represent a latent source of replication-competent virus capable of reestablishing infection upon treatment interruption. In this study, we demonstrated the presence of the latent macrophage reservoir in brains of SIV-infected ART-treated macaques and analyzed the reservoir using our established outgrowth assay to quantitate macrophages harboring replication-competent SIV genomes. Our results support the idea of the existence of other latent reservoirs in addition to resting CD4^+^ T cells and underscore the importance of macrophages in developing strategies to eradicate HIV.

## INTRODUCTION

Although the frequency of human immunodeficiency virus (HIV)-associated neurocognitive disorders (HAND) has decreased with the onset of antiretroviral therapy (ART), milder forms of neurologic impairment are still observed in virally suppressed HIV-infected individuals on ART (1–3). HAND is thought to be a result of chronic central nervous system (CNS) inflammation in the brain (1, 2, 4, 5). It is unclear whether inflammation is caused by incomplete penetration of ART into the CNS and persistent virus replication or whether brain macrophages (BrMΦ) harbor latent virus that reactivates, causing sporadic inflammatory responses (6). Indeed, some HIV-infected individuals on ART have no detectable virus in the plasma but have measurable levels of HIV RNA in the cerebrospinal fluid (CSF) (7, 8). In addition, HIV was detected after rebound in the CSF of patients in Boston, which had undetectable plasma HIV during ART interruption for several months (9). There is a continuing debate on the sources of virus in the CSF and the cause of the chronic inflammation in brain that leads to HAND.

Viral replication and activation of BrMΦ during infection contribute to the severity of CNS pathology in HIV-infected patients and in simian immunodeficiency virus (SIV) models of HAND (10–13). BrMΦ, which are the main targets of productive HIV infection in brain, include perivascular macrophages and microglia, and viral RNA can be detected in these cells by *in situ* hybridization (ISH) in HIV-infected patients (14, 15). Similarly, SIV RNA and DNA in SIV-infected macrophages can be detected throughout infection (16–21). Moreover, SIV DNA remains in the brain of infected pig-tailed macaques despite antiretroviral therapy (22). It is still unclear whether BrMΦ are latently infected and contain replication-competent virus.

Currently, the best-characterized latent reservoir is represented by resting CD4^+^ T cells (rCD4) (23, 24). Further research is needed to determine whether macrophages also fit the definition of a latent reservoir. Current strategies for HIV eradication are aimed at reactivating, immunologically eliminating, or excising integrated virus and thus reducing or eliminating the functional latent virus reservoirs (25, 26). However, these strategies, such as the use of T cell-activating cytokines (27), histone deacetylase (HDAC) inhibitors (28), phorbol esters that stimulate protein kinase C (PKC) activity (29, 30), and RNA-guided excision of integrated HIV (31), have been developed primarily to target rCD4s. Little is known about the effect of these approaches in latently infected macrophages or whether macrophages will require different strategies for elimination.

Measuring the reduction of the latent reservoirs is key in determining the efficacy of any strategy to eradicate HIV. For lymphocytes, the frequency of latently infected rCD4 in HIV-infected ART-treated individuals is best measured by the quantitative viral outgrowth assay (QVOA) (32, 33). Our laboratory members previously developed a similar QVOA to quantify latently infected rCD4 in SIV-infected ART-treated macaques that yielded frequencies similar to those observed in HIV-infected patients (34). However, macrophages have never been examined in a similar manner. In order to quantitate the frequency of productively infected macrophages, our laboratory members developed a quantitative viral growth assay similar to the rCD4 QVOA (35). Using the macrophage quantitative viral outgrowth assay (MΦ-QVOA), we demonstrated that monocytes from the blood and macrophages from bronchoalveolar lavage fluid, lungs, spleen, and brain of chronic SIV-infected pig-tailed macaques harbor replication-competent virus and showed that CD4^+^ T cells did not contribute to the quantitation of productively infected macrophages in the MΦ-QVOA. In addition, we demonstrated that brain of SIV-infected macaques that were virally ART suppressed for over 1 year harbored latent viral genomes that could be reactivated in an animal treated with latency-reversing agents (LRAs) and that the latent viral genomes were genetically distinct from virus in plasma (36). In this study, we expanded those studies in five SIV-infected macaques treated with ART, in addition to the three animals evaluated in the previous study. We used the MΦ-QVOA to quantitate the frequency of latently infected BrMΦ that produced replication-competent virus in suppressed animals. Latently infected macrophages were identified in 85.7% of ART-suppressed macaques. This report and our previous findings provide complementary data supporting the hypothesis that the brain represents a latent reservoir that harbors replication-competent virus. The results of these studies in the SIV model suggest that BrMΦ may provide another barrier to HIV eradication.

## RESULTS

### Variations of viral decay and set point in SIV-infected macaques treated with two different ART regimens.

We previously demonstrated that daily administration of tenofovir (TFV), atazanavir (ATV), saquinavir (SQV), and integrase inhibitor L000870812 (INTI) completely suppressed plasma and CSF viral loads to undetectable levels in an animal model for AIDS and HIV encephalitis that used pig-tailed macaques inoculated with SIV/DeltaB670 and SIV/17E-Fr (34). Using this model, which closely reproduces the neuropathological and immunological events identified in untreated and ART-treated HIV-infected patients (1,3), we demonstrated that (i) SIV infects the brain by 4 days postinfection (dpi), similarly to reports of HIV patients with HIV infection in brain in the first weeks (37, 38); (ii) virus genotypes in brain differ from those in the periphery in both the viral envelope and long terminal repeat (LTR) (39–41); (iii) virus replication in the CNS is differentially regulated in comparison to replication in the peripheral blood (13, 42–44); and (iv) tissue macrophages in spleen, lung, and heart harbor SIV-infected macrophages (45–48). In macaques treated with the four-drug regimen previously described (34), we observed biphasic viral decay in both plasma and CSF similar to the decay of HIV in ART-treated individuals. The frequency of latently SIV-infected rCD4 in blood and lymphoid tissue was very similar to the frequency observed in HIV-1-infected patients on long-term ART (ranging from 0.03 to 3 latently infected cells/per million rCD4) (23, 34, 49).

**FIG 1:**
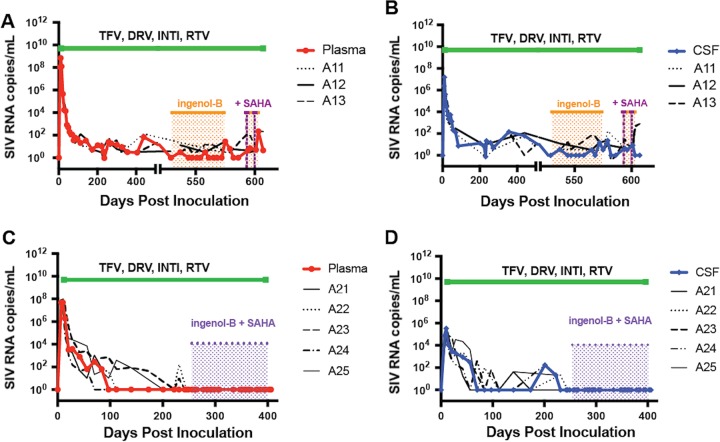
Viral load in plasma and CSF of two cohorts of SIV-infected ART-treated macaques. Two cohorts of SIV-infected pig-tailed macaques were treated with similar ART regimens (listed above the horizontal green line). (A and B) Macaques in cohort A1 were suppressed for more than 500 days and were treated with LRAs (ingenol-B, orange; ingenol-B plus suberoylanilide hydroxamic acid [SAHA], purple). (C and D) Macaques in cohort A2 followed similar protocol as those in cohort A1 but were suppressed for 100 to 400 days and treated with LRAs (ingenol plus SAHA in purple) in the last 60 days before euthanasia (Table 1). Median values for each group of animals are depicted in red for plasma (A and C) and dark blue for CSF (B and D). Analyses of samples with values below the limit of detection for the SIV qPCR assay (100 SIV RNA copies/ml) were repeated using ddPCR.

Efforts at reproducing this drug regimen in further studies have been less successful due to the lack of availability of SQV, which is no longer used in HIV-infected patients. Therefore, a similar drug combination was designed based on the CNS penetration effectiveness (CPE) rank system developed by Scott Letendre, in which a CPE score of 6 reflects a regimen with above-average effectiveness in the CNS (50). To maintain the same CPE rank of 6 used for our previous study, the protease inhibitors (PI) ATV and SQV were replaced by darunavir (DRV) plus the PI booster ritonavir (RTV) (21, 34). Eight pig-tailed macaques were dually inoculated with SIV/DeltaB670 and SIV/17E-Fr and were started on treatment with TFV, INTI, DRV, and RTV at 12 dpi. All animals were part of a study designed to evaluate the efficacy of LRAs in “shock and kill” strategies for HIV eradication and maintained viral suppression in plasma and CSF for 100 to 500 days. Three macaques in this group, henceforth called group A1, were virally suppressed for more than 500 days. Two of these macaques (PmA12 and PmA13) were treated with LRAs, while the third animal (PmA11) served as a control (36). After the second round of LRA, one of the LRA-treated macaques (PmA13) showed viral rebound in plasma and CSF without ART interruption (Fig. 1A and B). No viral reactivation was observed in the second LRA-treated macaque (PmA12) or the control animal (PmA11), indicating that those two macaques emulated the virological control of HIV-infected patients on long-term ART. The additional five macaques treated with the same ART regimen were virally suppressed for 100 to 400 days and were designated group A2 (Fig. 1C and D; Table 1). Similarly to group A1, two macaques (PmA22 and PmA23) in this group were treated with LRAs while the three other animals (PmA21, PmA24, and PmA25) served as a control group (36). In group A2, LRA treatment did not cause any detectable viral reactivation in either plasma or CSF and all five macaques had no increase in viral load.

**TABLE 1:** Description of macaque cohorts, the antiretroviral therapy regimen, and levels of CD4^+^ T cells, monocytes, plasma viral load, and CSF viral load at the terminal time point^a^

Cohort and animal identifier	ART (no. of days)	Cell counts (no. of cells/µl blood)		Viral load (no. of SIV RNA copies/ml)		Comment
		CD4^+^ T cells	Monocytes	Plasma	CSF	
A1						
PmA11	615	474	1,092	<10	<10	NA
PmA12	616	1,074	485	<10	<10	Treated with LRAs after 550 dpi
PmA13	605	320	1,346	1,800	18,000	Treated with LRAs after 550 dpi
						
A2						
PmA21	394	280	340	<10	<10	NA
PmA22	391	324	2,310	<10	<10	Treated with LRAs after 242 dpi
PmA23	345	331	410	<10	<10	Treated with LRAs after 249 dpi
PmA24	182	291	350	<10	<10	NA
PmA25	182	790	310	<10	<10	NA

^a^ The antiretroviral therapy (ART) regiment for both cohort A1 and cohort A2 consisted of tenofovir, darunavir, integrase inhibitor L000870812, and ritonavir. LRA, latency reverse agents; dpi, days postinfection; NA, not applicable.

**FIG 2:**
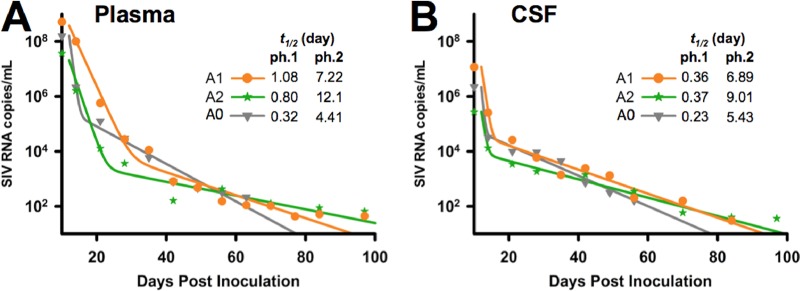
Analyses of viral decay in plasma and CSF. The line for each cohort represents the curve best fitting the geometric means of longitudinal viral loads (symbols) for that cohort in plasma (A) and CSF (B). Values in the insets show half-lives (*t*_1/2_) and *R*^2^ values for phase (ph) 1 and phase 2 in the curves. Cohort A0 represents data previously published (34).

Mathematical modeling of virus decay in plasma revealed distinct biphasic curves between our previous ATV/SQV regimen (34) and the new drug combination containing DRV/RTV (Fig. 2), indicating that the former combination was more efficacious with respect to the time to controlling and decreasing viral replication to undetectable levels. The observed differences in the half-life of virus decay in plasma, though, may not have been solely determined by the type of ART, as macaques in cohorts A1 and A2 were treated similarly but had distinct decay curves (*P* = 0.09 by *t* test comparing parameters fit to each animal; high interanimal variation increased the *P* values). Levels of CSF virus decay were similar among the three groups, with half-lives during the first decay phase shorter than those observed in plasma, suggesting that, during the first phase of ART, infected cells are more rapidly suppressed or eliminated in the CNS than in the periphery. The cause of the second decay phase in plasma has not been completely elucidated, but it has been conventionally attributed to the existence of longer-lived productively infected cells, in particular, tissue macrophages (51). Thus, to assess the potential contribution of BrMΦ to residual viral replication and, potentially, to a brain macrophage viral reservoir in the CNS during ART, we measured the number of productively infected macrophages isolated from all eight animals in the cohorts using a quantitative viral outgrowth assay that had been used previously to measure the number of SIV-infected macrophages in viremic animals (35).

### Quantitation of productively infected BrMΦ in ART-treated macaques.

The two predominant populations of mononuclear phagocytic cells in brain are microglia, which are derived from progenitor cells in the yolk sac, and perivascular macrophages, which are derived from circulating monocytes (52). Although the ratio between these two subsets varies during HIV/SIV infection and other CNS inflammatory changes, the majority of cells are resident microglia (53). Both types of cells are innate immune cells and produce type 1 interferon-stimulating gene products and cytokines in response to infection (43). To determine the number of productively infected BrMΦ in the CNS, mononuclear phagocytic cells from the A1 and A2 cohorts of SIV-infected ART-treated macaques were isolated and the frequency of cells that harbor replication-competent virus was determined by the macrophage quantitative viral outgrowth assay (MΦ-QVOA) as previously described (35). Although less than 5% of brain myeloid cells are perivascular macrophages (53), at least in untreated SIV-infected macaques they are the main source of replicative virus in brain in the CD8^+^ T cell-depleted SIV macaque model for HIV-associated CNS disease (10). Of note, our methodology cannot distinguish between microglia and brain macrophages and for the purposes of the results we refer to CD11b^+^ as brain macrophages (BrMΦ), fully aware that they almost certainly include distinct subpopulations. Distinguishing whether SIV latency is preferentially associated with some cellular subset must await future studies and the availability of more-specific reagents for macaque cells.

**FIG 3:**
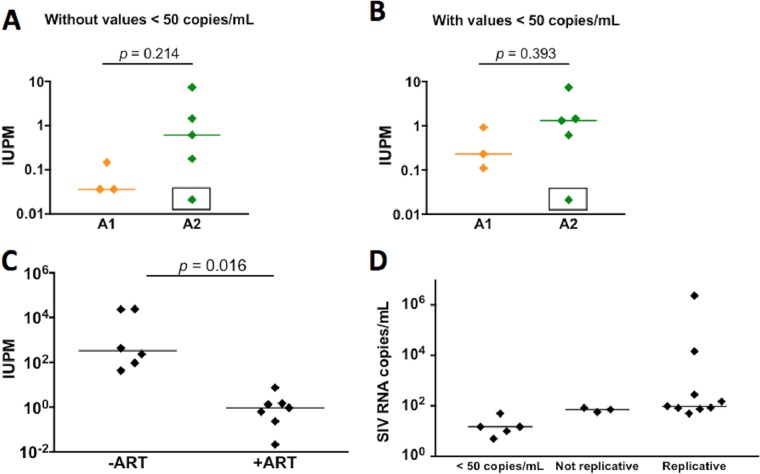
Quantitation of latently infected BrMΦ in ART-treated macaques by MΦ-QVOA. (A and B) Quantitation of infected BrMΦ from two groups of ART-treated macaques. In panel B, wells containing <50 SIV RNA copies/ml were also considered positive, and IUPM (infectious units per million cells) values were calculated accordingly. Boxes in panels A and B indicate values under the limit of detection, i.e., BrMΦ samples that did not present positive well results by qPCR. (C) Comparison between the numbers of SIV-infected BrMΦ isolated from animals that were not given ART (−ART) and the numbers isolated from animals that were treated with ART and showed full suppression (+ART; PmA13 not included). The horizontal black line represents the median IUPM values. The MΦ-QVOA results from SIV-infected animals without ART were previously reported (35). Significance was determined by Mann-Whitney nonparametric *t* test; a *P* of <0.05 was considered significant. (D) Numbers of SIV RNA copies in the supernatants of QVOA-positive wells separated according to the pattern of replication (<50 copies/ml, >50 copies/ml without spread, or replicative).

For the QVOA, BrMΦ isolated from infected macaques were serially diluted, plated in duplicate or triplicate wells, and cocultured with CEMx174 cells to expand the infectious virus released from the BrMΦ. After 13 days, SIV RNA was quantitated in the supernatants by quantitative PCR (qPCR). Wells containing more than 50 RNA copies/ml (the qPCR limit of detection is 10 SIV RNA copies per reaction) were considered to represent a positive result, and the number of latently infected cells that contained replication-competent virus was determined using an algorithm for maximum likelihood estimation of IUPM (54). In seven of eight macaques (87.5%), the QVOA measured BrMΦ harboring replication-competent SIV genomes, with levels of latently infected cells ranging from 0.11 to 7.36 IUPM (Fig. 3A and B; Table 2). No significant difference between the two cohorts in the number of infected BrMΦ was observed, regardless of the duration of ART treatment. In addition, no significant difference was observed when we considered wells that contained between 5 and 50 SIV RNA copies/ml to represent a positive result (Fig. 3A and B). BrMΦ QVOA IUPM values also correlated poorly with levels of blood CD4^+^ T cells (*R* = −0.2143, *P* = 0.6191) and monocytes (*R* = −0.4286, *P* = 0.2992) quantitated at necropsy. The number of productively infected BrMΦ measured by QVOA in the SIV-infected ART-treated macaques was significantly lower than that measured in the untreated SIV-infected animals (IUPM median of 0.268 versus 231, *P* < 0.0001) (Fig. 3C). Our group previously observed a high correlation between viral load in CSF and levels of SIV RNA in brain tissue from SIV-infected ART-naive macaques (17, 55), and similar results were observed in suppressed macaques in this study. The only macaque with detectable SIV RNA in brain and CSF was PmA13, which also had plasma viremia after LRA treatment and, therefore, was not virally suppressed at necropsy. All other animals had undetectable SIV RNA in brain tissue and CSF independently of the number of infected BrMΦ quantitated by QVOA. No infected BrMΦ were found in macaque PmA24 QVOA, suggesting either that some animals in our model did not harbor latent reservoirs in the brain or that the BrMΦ QVOA is not sensitive enough to identify positive cells in all macaques due to sampling of brain. It has been reported that SIV infection in CNS is highly focal; therefore, a larger number of cells may have to be used in the QVOAs for a more sensitive evaluation. Regardless, these data provide evidence that macrophages can serve as latent reservoirs for SIV.

**TABLE 2:** SIV DNA and RNA levels in two brain regions (basal ganglia and parietal cortex) at terminal point and number of latently infected BrMΦ for each macaque^a^

Cohort and animal identifier	No. of SIV DNA copies per 10^6^ cells		No. of SIV RNA copies per µg RNA		Brain MΦ-QVOA IUPM	
	Basal ganglia	Parietal cortex	Basal ganglia	Parietal cortex	Without wells (<50 copies/ml)	With wells (<50 copies/ml)
A1						
PmA11	<10	<10	<10	<10	0.147	0.917
PmA12	<10	<10	<10	<10	0.036	0.230
PmA13	<10	64	<10	<10	0.036	0.110
						
A2						
PmA21	<10	<10	<10	<10	1.445	1.445
PmA22	<10	<10	<10	<10	0.177	1.309
PmA23	<10	<10	<10	<10	7.362	7.362
PmA24	<10	<10	<10	<10	<0.021	<0.021
PmA25	<10	<10	<10	<10	0.613	0.613

^a^ QVOA values are presented and represent the presence or absence of positive wells with fewer than 50 SIV RNA copies/ml of supernatant. Values reflect maximum likelihood estimates of infection frequency in IUPM (infectious units per million cells). Given the resolution of the assay, the 95% confidence interval is typically 0.2 to 4 times the reported value.

This conclusion is supported by our results measuring levels of T cell receptor beta gene (*TCR*β) RNA in the QVOA assay and demonstrating negligible levels of CD4^+^ T cells in BrMΦ cultures (Table 3). Indeed, judging on the basis of the frequency of infected CD4^+^ T cells in the blood estimated by QVOA (34), the number of potentially infected CD4^+^ T cells present in the CD11b-selected BrMΦ that we calculated in the MΦ-QVOA was less than 1 (Table 3).

**TABLE 3:** Quantitation of infected CD4^+^ T cells in the MΦ-QVOA using detection of *TCR*β RNA, as described by Avalos et al. (35)

Cohort and animal identifier	*TCR*β in MΦ-QVOA (no. of RNA copies/10^5^ cells)	% CD3^+^ T cells in MΦ-QVOA by TCRβ	% CD4^+^ T cells in blood CD3^+^ T cells	% CD4^+^ T cells in MΦ-QVOA	CD4^+^ T cell IUPM by PBMC QVOA	No. of infected CD4^+^ T cells per 10^6^ QVOA cells
A1						
PmA11	3,871	0.97	50.9	0.50	1.3	0.0065
PmA12	0	0	60.2	0	0.30	0
PmA13	20,428	5.11	35.1	1.80	0.40	0.0072
						
A2						
PmA21	0	0	55.2	0	0.05	0
PmA22	0	0	56.8	0	0.07	0
PmA23	0	0	56.4	0	0.05	0
PmA24	0	0	45.8	0	0.01	0
PmA25	0	0	48.9	0	0.06	0

### Different patterns of viral replication in BrMΦ QVOA wells.

There are major differences between the CD4^+^ T cell QVOA and the macrophage QVOA. First, CD4^+^ T cells proliferate during the assay and clonal expansion probably contributes to increased sensitivity in detecting viral particles in the QVOA. Macrophages do not proliferate *in vitro*, although there is no loss of cells during the assay and the morphology and function are maintained for more than 2 weeks (35). Second, viruses adapted to efficient replication in myeloid cells in brain may not optimally expand in the cell lines used for the assay (34, 56). Therefore, the presence of QVOA wells containing low levels of RNA (i.e., <50 SIV RNA copies/ml or 10 copies/reaction) may be significant in evaluating BrMΦ latent reservoirs for “shock and kill” strategies since low levels of potentially replication-competent latent genomes could contribute to virus rebound.

Detection of replication-competent virus in the CD4^+^ T cell QVOAs in suppressed animals (34) and in MΦ-QVOAs from nonsuppressed macaques (35) is consistent with virus reactivation and sustained replication. However, efficacious viral replication was not always observed in the BrMΦ QVOAs from virally suppressed macaques. In these assays, we observed three distinct temporal patterns of viral replication. In the majority (53%) of positive QVOA wells, levels of viral RNA in the supernatant increased in samples collected at 5 to 7 days and 10 to 13 days postcoculture (dpc), indicating viral spread and *de novo* infection (Fig. 3D). In 17% of positive wells, we observed a lower number (50 to 100 copies/ml) of SIV RNA that did not increase in the supernatant samples obtained at 5 to 7 and at 10 to 13 dpc, suggesting that viral particles were constantly released from the cells but that there was no viral expansion in CEMx174 cells. A third pattern was observed in 30% of positive wells, where fewer than 50 SIV RNA copies/ml were detected in pooled supernatants after 10 to 13 dpc. In some cases, SIV RNA was detected at one time point but not at previous or subsequent time points, corroborating the hypothesis that SIV latency and reactivation in macrophages are modulated by a mechanism distinct from that seen with CD4^+^ T cells. Thus, while we have demonstrated that BrMΦ contain replication-competent virus after reactivation, high levels of virus replication are not always detected in the BrMΦ QVOA assay. The inclusion of wells with sporadic release of low levels of viral particles (Fig. 3B) did not alter our calculation of the number of BrMΦ samples considered infected (87.5%) but increased the levels of infected cells per sample in half of the analyzed macaques. These changes, however, were not significant since they fell within the 95% confidence interval, which, given the resolution of the assay, was typically 0.2 to 4 times the reported value.

### SIV virions released by BrMΦ are replication competent.

**FIG 4:**
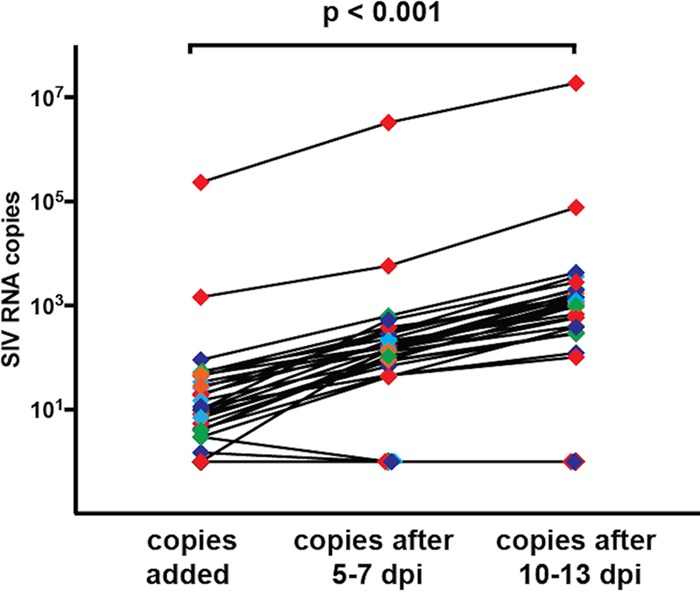
Virus from BrMΦ QVOA is replication competent in PBMCs. The graph depicts the increased levels of SIV RNA in supernatants of PBMC cultures that were subjected to spinoculation with 100 µl of supernatants collected from BrMΦ QVOA wells. Different wells from each of the five analyzed macaques are depicted in the same color, and each line represents the result of one supernatant transfer. The first time point depicts the number of SIV RNA copies contained in the 100-µl volume of the BrMΦ QVOA well that was used for spinoculation. The other time points show the numbers of total supernatant copies measured at 5 to 7 days and 10 to 14 days postspinoculation.

Because it was not clear whether strains of SIV in BrMΦ were capable of optimal replication in CEMx174 cells, primary macaque peripheral blood mononuclear cell (PBMC) cultures were used to assess the infectivity of viruses present in QVOA wells. Thus, 100 µl of supernatants collected from BrMΦ QVOAs was transferred to phorbol myristate acetate/phytohemagglutinin/interleukin-2 (PMA/PHA/IL-2)-activated uninfected macaque PBMCs and the reaction mixture was subjected to spinoculation for 2 h. Results of quantitation of SIV RNA from the MΦ-QVOA supernatants (input virus) and from infected PBMCs (output virus) are shown in Fig. 4. Replication of virus was observed in most BrMΦ QVOA samples tested, including in PBMCs subjected to spinoculation with supernatants from some samples that contained fewer than 50 SIV RNA copies/ml. In contrast, supernatants from wells in which no SIV RNA was detected during the QVOA were also tested and no virus infection was observed. These results indicate that, as a consequence of considering wells with more than 50 SIV RNA copies/ml to represent positive results, our BrMΦ QVOAs underestimate the size of the latent reservoir in brain myeloid cells. Nonetheless, these results corroborate our previous findings indicating that BrMΦ harbor long-term latent virus genomes that can contribute to plasma and CSF viremia once ART is withdrawn (36).

### BrMΦ harbor SIV latent genomes that can be reactivated.

The mechanisms that establish or maintain HIV or SIV in a latent state in macrophages have not been fully elucidated. Nonetheless, our results demonstrate that BrMΦ harbor replication-competent, transcriptionally silenced viral genomes *in vivo* that can be activated *in vitro* in the MΦ-QVOAs. Analyses of total SIV RNA isolated from brain (basal ganglia and parietal cortex) show that there were no detectable quantities of SIV RNA (>10 copies per µg of tissue) in 7 of the 8 macaques. Only LRA-treated animal PmA13, which also had CSF and plasma viremia and therefore was not virally suppressed, had detectable viral RNA in brain. The seven animals had no detectable SIV RNA in brain tissues, suggesting that the BrMΦ used in the QVOAs were not transcribing viral RNA prior to *in vitro* cultures. To confirm that the isolated cells used for QVOAs had latent SIV genomes, SIV DNA and RNA from CD11b^+^ cells were quantitated for the BrMΦ cultures by Droplet Digital PCR (ddPCR) (Table 4). All animals with quantifiable levels of SIV DNA in brain tissues also had SIV DNA in CD11b^+^ sorted cells. In LRA-treated animal PmA13, in addition to 119 SIV DNA copies/10^6^ sorted cells, we observed a high level of SIV RNA expression (33,984 SIV RNA copies/10^6^ sorted cells), suggesting that BrMΦ contributed to the level of virus detected in the CSF during LRA treatment. All seven fully suppressed animals also had SIV DNA genomes in CD11b^+^ sorted cells, and three had BrMΦ transcribed SIV RNA prior to cell plating. The quantity of SIV RNA detected in the CD11b^+^ cells did not correlate with the number of cells that produced replication-competent SIV in the BrMΦ QVOA. This suggests that the level of RNA expression in cells may not reflect transcription from replication-competent virus in BrMΦ and that activation of viral RNA may occur without the production of infectious virus.

**TABLE 4:** SIV DNA and RNA levels in BrMΦ isolated from basal ganglia and parietal cortex of SIV-infected ART-treated macaques^a^

Cohort and animal identifier	SIV DNA (no. of copies per 10^6^ BrMΦ)	SIV RNA (no. of copies per 10^6^ BrMΦ)	CSF (no. of SIV RNA copies/ml)	Basal ganglia (no. of SIV RNA copies/µg total RNA)	MΦ-QVOA (no. of infected BrMΦ per million cells)
A1					
PmA11	20	<5	<10	<10	0.92
PmA12	9	<5	<10	<10	0.23
PmA13	119	33,984	18,000	<10	0.11
					
A2					
PmA21	40	<5	<10	<10	1.45
PmA22	60	<5	<10	<10	1.31
PmA23	1,606	230	<10	<10	7.36
PmA24	57	4,652	<10	<10	<0.02
PmA25	67	1,263	<10	<10	0.61

^a^ SIV RNA levels in CSF and basal ganglia and no. of infected BrMΦ evaluated at necropsy are also presented for comparison.

Overall, our results corroborate the assertions that the BrMΦ QVOA underestimates the size of the virus reservoir and that infection in the brain is highly focal and is not evenly distributed in the tissue such as has been observed in viral infection in CD4^+^ T cells in the blood. In addition, HIV *gag* RNA has been observed in rCD4s isolated from HIV-infected suppressed patients and may represent defective or hypermutated genomes that do not produce viral proteins. Nonetheless, four of seven animals had no SIV RNA in CD11b^+^ selected cells before plating, indicating that the virus in the supernatant of BrMΦ cultures was derived from previously latent genomes.

### Detection of SIV RNA-expressing cells by ISH in brain.

**FIG 5:**
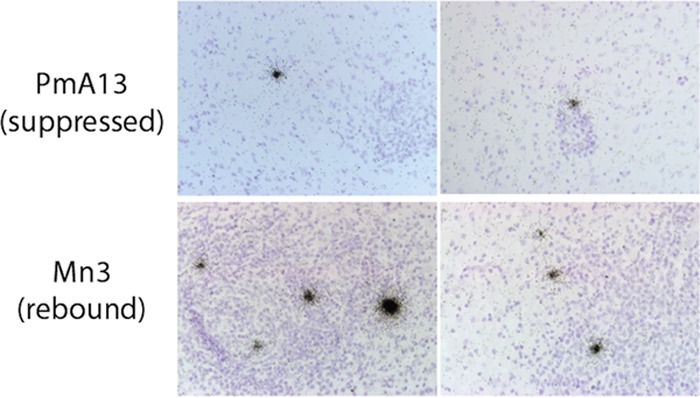
*In situ* hybridization (ISH) for SIV RNA in brain sections. Data are representative of ISH results of analysis of SIV RNA in brain (occipital cortex) of macaque PmA13 (top row) and Mn3, which represents a previously published SIV-infected macaque that discontinued ART and had SIV rebound (lower row) (36); magnification, ×40.

ISH was performed in two regions of brain to investigate the frequency and pattern of SIV cell-associated RNA (caRNA) in regions of the brain. In addition, ISH complemented the QVOA results as another quantitative technique *in vivo*. In cohort A1, levels of virus in macaques were suppressed for over 500 days and the macaques remained on ART while being treated with LRA, during which time macaque PmA13 had reactivation of virus in CSF (Fig. 1). Brain sections (occipital cortex) from macaques PmA11, A12, and A13 were also analyzed by ISH. RNA-positive cells were found in the occipital cortex of macaque PmA13 (Fig. 5; Table 5) at a density of 2,220 SIV^+^ cells per gram of tissue, whereas those in PmA11 had 10-fold-lower density (202 SIV^+^ cells/g of tissue) in occipital cortex. In both macaques, the pattern of positive cells *in situ* consisted of a single positive focus of RNA detected per analyzed area, suggesting that expression of SIV RNA in the brain occurred without viral spread to other cells. Macaque PmA12 had no detectable SIV RNA^+^ cells in the areas analyzed.

**TABLE 5:** Number of SIV RNA-positive foci per gram of brain tissue estimated by *in situ* hybridization

Cohort and animal identifier	Area analysed (μm^2^)	No. of positive foci per analysed field	Density of SIV^+^ cells per gram of tissue
A1			
PmA11	1.41 × 10^8^	0, 1, 0, 0, 0, 0, 0	202
PmA12	2.54 × 10^8^	0, 0, 0, 0, 0, 0, 0	<102
PmA13	1.16 × 10^8^	1, 1, 1, 1, 1, 3, 1	2,220

ISH of SIV RNA in brain and the BrMΦ QVOA provide very different quantitative approaches to detecting RNA-expressing infected cells. While ISH analyzes multiple thin sections from brain regions and quantitates cell expression of cell-associated SIV RNA, the QVOA detects replication-competent virus in isolated cells. Our group routinely isolates BrMΦ cells from basal ganglia, thalamus, and frontal, parietal, and temporal cortices, leaving other brain regions, such as occipital cortex, for ISH and immunohistochemistry. Results of QVOA of SIV-infected macrophages isolated from basal ganglia were positive despite the absence of detectable SIV RNA by qPCR observed in the same section (Table 2), suggesting that there are far more cells in the brain harboring SIV genomes that do not express viral RNA. Thus, these complementary quantitative techniques support the idea that BrMΦ in brain of SIV-infected ART-treated macaques harbor latent viral genomes and represent a functional viral reservoir.

## DISCUSSION

In the era of ART, signs of chronic systemic inflammation are observed in a large number of patients, even when blood viral load is undetectable and CD4^+^ T cell counts are restored to preinfection levels (57, 58). HAND remains prevalent in ART-treated patients along with elevated markers of neuronal damage and macrophage inflammation in the CSF (1, 2, 59), but the issue of the presence of functional latent reservoirs in the brain remains controversial. Using our SIV macaque model for AIDS and HIV encephalitis, we recently demonstrated that the brain of SIV-infected macaques virally suppressed for more than 500 days harbored latent SIV genomes that were reactivated during treatment with the protein kinase C (PKC) agonist ingenol-B and the HDAC inhibitor vorinostat (36). SIV RNA detected by ISH colocalized with CD68^+^ cells, indicating that macrophages in the brain were productively transcribing viral RNA after LRA treatment. In this study, we quantitated the number of macrophages that harbor replication-competent virus in brain during ART suppression.

Most animals in the study harbored latently infected macrophages in regions of the brain that contained no detectable viral RNA. Evaluation of samples enriched for CD11b^+^ BrMΦ, however, led to the detection of SIV *gag* transcripts in three of seven fully virally suppressed animals, suggesting that not all cells used for the MΦ-QVOA were transcriptionally silent in the context of SIV gene expression. Similar results are observed in CD4^+^ T cells isolated from HIV^+^ patients with long-term viral suppression, indicating that both cell populations contain both RNA-producing viral genomes and fully suppressed genomes that have the potential to be reactivated *in vitro* (60–62). The presence of cell-associated RNA does not indicate infectious virus production, since, at least in rCD4s, most integrated viral genomes are defective or hypermutated (63). Similar events may occur in BrMΦ, as preplated CD11b^+^ cells from animal PmA24 had more than 4,000 SIV RNA copies/million cells but did not produce viral particles once seeded and activated (Table 4). Conversely, SIV RNA was undetectable by ddPCR in CD11b^+^ BrMΦ from four macaques despite the detection of viral RNA in the wells of QVOAs from the same animals. The virus released from BrMΦ was infectious in primary macaque PBMCs.

Furthermore, SIV-infected macaques that were virally suppressed for 6 months or more with little or no detectable virus in the CSF and low levels of SIV RNA in brain also had latently infected BrMΦ that produced virus in the QVOA. After 1.7 years of viral suppression, macaques in cohort A1 showed no viral RNA in basal ganglia and parietal cortex. Nevertheless, all three macaques had replication-competent virus produced in the isolated BrMΦ. Of note, we detected viral RNA by ISH in the brain of LRA-treated macaque PmA13, but the viral RNA was detected in the occipital cortex, a brain section not used for the BrMΦ QVOA. These results corroborate findings showing that SIV and, potentially, HIV infection in the brain is highly focal (36) and can provide variable results depending on the brain region analyzed for each specific assay.

Our data also show that the mechanisms controlling viral expression in BrMΦ are somewhat distinct from those in CD4^+^ T cells and warrant further investigation. QVOAs of resting CD4^+^ T cells provided a unique approach to quantifying and understanding the mechanisms of SIV and HIV latency in lymphocytes (32, 34, 63). With the establishment of the Mø-QVOA, the assay will provide a way to examine the establishment of latency and the mechanisms of reactivation in myeloid cells. In this study, we identified latently infected BrMΦ in brain samples containing fewer than 10 copies of SIV DNA per million cells. Indeed, in animals virally suppressed for more than 500 days, the number of infected macrophages measured in the Mø-QVOA ranged from 3.6 to 15 per hundred million cells, supporting the results showing the low level of DNA detected by qPCR. Thus, the quantitation of SIV DNA or RNA by PCR in brain tissue does not fully reflect the size of the latent functional reservoir, which is the main target in eradication strategies. In addition, the results from the Mø-QVOA showing that a small number of replication-competent viruses are sporadically released in some isolates of latent BrMΦ indicate that the parameters that we used to define a positive QVOA well (>50 copies/ml after 10 to 13 dpc) underestimate the number of latently infected cells that produce replication-competent virus. Thus, refinement of the assay using primary PBMCs would likely demonstrate a higher level of functional latency in BrMΦ in brain.

The demonstration that there is latent replication-competent virus in SIV-infected ART-suppressed macaque brain provides a mechanism for the ongoing macrophage activation observed both in the macaques and in HIV individuals virally suppressed on ART. Recent studies have suggested that, while virus does not spread during ART suppression, there is ongoing stochastic activation of virus genomes in latently infected cells (64, 65). Reactivation of virus without spread in the macrophage is likely to induce innate immune responses and cellular activation. Thus, the presence of productively infected latent MΦ in the brain provides a mechanism for the ongoing inflammation of HIV in a fully virally suppressed individual. Also, it has been recently demonstrated that defective provirus expressed in rCD4s can be recognized by adaptive immune responses, shaping the proviral landscape (66). It is possible that similar responses can happen with viral proteins generated from defective proviruses in BrMΦ.

The SIV model used in this study included viruses with tropism for both CD4^+^ T cells and macrophages; therefore, this study may not be directly comparable to studies of other phenotypically restricted animal models or HIV-infected subjects. The detection of virus in the CSF of virally suppressed patients (7, 8) is a strong indication of the presence of HIV-infected cells in human brain. However, precise quantitation of viable, blood-free, infected brain cells from human patients remains difficult despite the availability of well-characterized HIV brain from the National NeuroAIDS Tissue Consortium (NNTC; supported by the National Institute of Mental Health [NIMH]). Procedural obstacles, such as those represented by time of sample acquisition and blood CD4^+^ T cell contamination due to lack of perfusion, complicate the interpretation of results. Our model and the BrMΦ QVOA provide an experimental protocol for quantitating and understanding mechanisms of viral latency in brain, as well as for examining the establishment and role of viral reservoirs in the CNS.

The presence of a long-term functional reservoir of SIV in brain that parallels the biological and pathological features of infected individuals with HIV encephalitis suggests that the presence of HIV in brain may be a formidable barrier to strategies designed to decrease or eliminate the reservoir. First, the brain is protected by the blood-brain barrier; therefore, eradication approaches should take into account strategies for CNS penetration. However, strategies that include activation of virus in brain may have the effect of increasing inflammation and neuronal toxicity due to increased macrophage activation and production of cytokines, as we observed in a virally suppressed macaque treated with two cycles of LRAs (36). Conversely, if the brain is excluded from eradication strategies, there is the potential for incomplete elimination of a functional latent reservoir that can be reactivated when ART is stopped, resulting in the spread of reactivated CNS virus to peripheral blood and tissues. Our study results strongly suggest that the brain harbors a functional latent SIV reservoir and that BrMΦ in the CNS should be considered for the complete elimination of latent HIV in AIDS cure strategies.

## MATERIALS AND METHODS

### Ethics statement and animal studies.

Animal studies were conducted in accordance with the *Weatherall Report*, the *Guide for the Care and Use of Laboratory Animals*, and the USDA Animal Welfare Act. All studies were approved by the Johns Hopkins University Institutional Animal Care and Use Committee under protocol PR12M310. Eight juvenile male pig-tailed macaques (*Macaca nemestrina*) were intravenously inoculated with SIV/DeltaB670 and SIV/17E-Fr as previously described (13). Animals were monitored twice daily by trained technicians or veterinarians for clinical signs of disease, such as weight loss, intractable diarrhea, and opportunistic infection, so that early termination (endpoint) could be performed if necessary. Macaques were housed in Johns Hopkins University facilities, which are fully accredited by the Association for the Assessment and Accreditation of Laboratory Animal Care International (AAALAC International), and fed a balanced macaque chow (Purina Animal Nutrition, Gray Summit, MO). All animals were housed in groups prior to infection and in cages providing 6 sq ft of space with environmental enrichment (manipulanda and novel foodstuffs) and also visual and auditory contact of conspecifics. As mandated by the USDA, animals were housed in pairs following infection, which helped ensure that animals received the appropriate drug dosage. Macaques were treated 12 days postinoculation (dpi) with 30 mg/kg of body weight for the first 2 weeks and then 10 mg/kg of tenofovir (TFV; Gilead, Foster City, CA) once daily intramuscularly (i.m.) as previously described (34), 480 mg/kg of darunavir (DRV; Janssen, Titusville, NJ) orally twice daily, 10 mg/kg of the integrase inhibitor L000870812 (INTI; Merck, Kenilworth, NJ) orally twice daily, and 24 mg/kg of ritonavir (RTV; AbbVie, North Chicago, IL) orally twice daily. Necropsies were scheduled at prespecified time points ranging from 157 days to 616 days post-ART initiation. Blood and cerebrospinal fluid (CSF) samples were collected longitudinally. For necropsy, animals were euthanized using sodium pentobarbital while under ketamine sedation (10 mg/kg intramuscular injection) prior to perfusion with phosphate-buffered saline (PBS) to remove blood from tissues as previously described (13).

Viral loads in plasma and CSF, CD4^+^ T cell counts in blood, and tissue RNA and DNA levels in parietal cortex and basal ganglia were determined for all macaques in the study (Table 1). These studies were performed in accordance with federal guidelines and institutional policies.

### Isolation of brain macrophages by CD11b selection.

All animals were perfused before necropsy to remove blood from tissues. Brain sections from regions that have been shown to support SIV replication (67), i.e., basal ganglia, thalamus, and frontal, parietal, and temporal cortices, were physically stripped of meninges and ventricles, minced using a scalpel, and washed twice with PBS. Tissue was then digested for 30 min in trypsin-DNase digestion solution (Dulbecco’s modified Eagle medium [DMEM] supplemented with 0.25% trypsin, 50 µg DNase/ml, and 50 µg gentamicin/ml) at 37°C with agitation. Digested tissue was filtered twice, first through a 183-µm-pore-size sterile mesh and then through a 100-µm-pore-size sterile filter, which was then washed once with DMEM–10% fetal bovine serum (FBS) (DF10) to maximize the number of collected cells. The flowthrough was centrifuged for 10 min at 400 × *g*, and pelleted cells were washed twice with DF10 before being resuspended in 36 ml of PBS. The cell suspension was then gently mixed with 9 ml Percoll in a 50 ml round-bottom open-top polypropylene tube (Nalgene) and ultracentrifuged at 411,000 × *g* for 30 min at room temperature. BrMΦ were harvested from the gradient layer below the myelin cap using a syringe connected to a three-way stopcock and then filtered through a 40 µm-pore-size mesh to remove capillaries. DF10 was used to rinse the mesh and also to increase the total cell suspension volume to 35 ml. Cells were pelleted for 10 min at 400 × *g*, resuspended in 10 ml DF10, and counted by the use of a Muse cell analyzer (Millipore).

BrMΦ were positively selected from the cell suspension using a fluorescein isothiocyanate (FITC)-conjugated anti-CD11b antibody (clone BEAR1; Beckman Coulter, Inc., Brea, CA) followed by the use of an EasySep FITC positive-selection kit (StemCell Technologies) (35). After selection, a subset of BrMΦ were costained with CD3 (clone SP34-2; BD Biosciences) and a LIVE/DEAD fixable dead cell stain kit (Invitrogen) was used to evaluate selection purity by flow cytometry using an LSRFortessa cell analyzer (BD Biosciences). CD11b^+^ purified macrophages were resuspended in 5 ml of BrMΦ media (DMEM [Life Technologies, Inc.] supplemented with 5% heat-inactivated FBS [Atlanta Biologicals], 5% IS giant cell tumor conditioned media [Irvine Scientific, Santa Ana, CA], 100 U/ml penicillin-streptomycin [Life Technologies, Inc.], 70 μg/ml gentamycin [Life Technologies, Inc.], 2 mM l-glutamine [Life Technologies, Inc.], 3 mM sodium pyruvate [Sigma], and 10 mM HEPES buffer [Life Technologies, Inc.]) for QVOAs. An extra aliquot of 10^6^ BrMΦ was snap-frozen for DNA and RNA isolation.

### Brain macrophage quantitative viral outgrowth assay (MΦ-QVOA).

CD11b^+^ BrMΦ were resuspended in BrMΦ media for a final concentration that ranged from 2.5 × 10^5^ to 2.5 × 10^6^ cells/ml, depending on the yield of cells in isolation from each macaque. Cells were serially diluted 10-fold in the presence of 10 μM zidovudine (Sigma), 25 nM DRV (Janssen), and 5 nM RTV (Merck) and plated on poly-l-lysine-coated plates. Plates were spun at 400 × *g* for 10 min to splatter cells and increase adherence. For wells containing 10^6^ to 10^7^ cells per well, we used 6-well plates to culture duplicates in 4 ml media. Higher serial dilutions (10^5^ to 10 cells/well) were plated in 24-well plates to maintain cell density similar to that represented by the 10^6^ to 10^7^ cells per well. Two extra wells with 10^5^ cells/well (TCR control wells) were plated using 24-well plates to evaluate CD3^+^ T cell contamination by *TCR*β RNA analysis. After a 3-day incubation to maximize adherence to the plates, antiretroviral-containing medium was removed and cells were briefly treated with 0.05% trypsin to remove any residual CD3^+^ lymphocytes that could be weakly attached to macrophages. After trypsinization, cells were rinsed once with PBS and replenished with BrMΦ activation media containing 10 ng/ml tumor necrosis factor alpha (TNF-α; ProSpec), 1 μg/ml Pam3csk4 (Sigma), and 1 μg/ml prostaglandin (Sigma) (used to reactivate latently infected macrophages). In addition to cell activators, 1 × 10^5^ CEMx174 feeder cells were added to each well to promote viral expansion and increase the assay sensitivity. TCR control wells were kept without feeder cells throughout the experiment. Supernatants were collected at 5, 7, 10, and 13 days postcoculture (dpc). For each supernatant collection, an equal volume of fresh BrMΦ activation medium was added. To determine the presence of viral particles, supernatants from 5 and 7 dpc and 10 and 13 dpc were pooled for RNA isolation and SIV RNA quantitation by quantitative reverse transcriptase PCR (qRT-PCR). The frequency of cells harboring replication-competent virus was determined using the IUPMStats v1.0 infection frequency calculator, and the data were expressed as infectious units per million (IUPM) (54).

### Quantitation of CD3^+^ T cells in MΦ-QVOA wells by the assessment of *TCR*β RNA levels.

TCR control wells without CEMx174 cells were used for *TCR*β RNA analyses. After 13 days in culture, BrMΦ were lysed for total RNA isolation. Absolute numbers of CD3^+^ T cells in MΦ-QVOA wells were estimated using a qRT-PCR assay for *TCR*β RNAs, which are uniquely presented in lymphocytes (35). For normalization, quantitation of 18S rRNA was performed in multiplex with the *TCR*β RNA by qPCR using primers and probes previously described (37). To estimate the number of infected CD4^+^ T cells that could potentially be found in MΦ-QVOA wells, levels of latently infected CD4^+^ T cells in blood were assessed by QVOA as previously described (34, 35).

### RNA and DNA isolation from cells and tissues.

RNA and DNA from cell cultures and sections from brain tissue were isolated by the use of an AllPrep DNA/RNA minikit (Qiagen, Valencia, CA) according to the manufacturer’s protocol with modifications. An on-column DNase digestion was performed on the RNA isolation column using an RNase-free DNase kit (Qiagen) with the addition of 4 units of TURBO DNase (Life Technologies, Inc.) to the enzyme mix.

SIV RNA from plasma, CSF, and culture supernatants was isolated using a QIAamp MinElute Virus Spin kit (Qiagen) and 200 μl of sample or a QIAamp MinElute Virus Vacuum kit (Qiagen) and 1 ml of sample, according to the manufacturer’s protocol, with modifications. An on-column DNase digestion was performed using an RNase-free DNase kit (Qiagen), with the addition of 3 units of RQ1 DNase (Promega, Madison, WI) to the enzyme mix.

RNA from frozen tissues was isolated with RNase STAT-60 (Tel Test Inc., Friendswood, TX). Tissues were homogenized using a FastPrep-24 instrument (MP Biomedicals, Santa Ana, CA) and lysing matrix D tubes (MP Biomedicals). After the addition of chloroform, samples were centrifuged and the aqueous phase was transferred to another tube for RNA precipitation with isopropanol. RNA was purified with an RNeasy minikit (Qiagen), with on-column DNase digestion performed using an RNase-free DNase kit (Qiagen) and the addition of 3 units of RQ1 DNase (Promega) to the enzyme mix.

DNA from frozen tissues was isolated with a DNeasy Blood and Tissue kit (Qiagen) according to the manufacturer’s protocol with modifications. Tissues were homogenized with a FastPrep-24 instrument (MP Biomedicals) in lysing matrix A tubes (MP Biomedicals). Samples were incubated with 100 mg/ml RNase A prior to DNeasy isolation.

### Quantitation of SIV RNA and DNA.

All SIV RNA levels were measured by qRT-PCR using a QuantiTect Virus kit (Qiagen) and primers for SIV *gag* as previously described (68, 69). Three reactions were performed for each sample. To control for DNA contamination, one reaction was analyzed without reverse transcriptase. For plasma and CSF, all samples with fewer than 1,000 SIV RNA copies/ml were reassayed by ddPCR, as previously described (36).

SIV DNA was measured by qPCR using an MP kit (Qiagen) and primers for SIV *gag* as previously described. Two reactions were performed for each sample and multiplexed with primers for the quantitation of beta interferon (IFN-β). The number of cells per reaction was calculated by quantitation of IFN-β DNA (two copies per cell) and used to normalize cellular SIV DNA and RNA levels from the same isolate. Samples were analyzed using a Rotor-Gene thermocycler (Qiagen).

### *In situ* hybridization (ISH).

Eight-micrometer-thick sections from fresh frozen brain (occipital cortex) were embedded in OCT, cryosectioned, postfixed in 4% paraformaldehyde, and dehydrated through the use of graded ethanols. Tissue sections were rehydrated through the use of graded ethanols and treated with HCl, triethanolamine, digitonin, and 4 μg/ml proteinase K. After acetylation with acetic anhydride and dehydration, samples were hybridized at 45°C overnight with a 35S-labeled riboprobe and 0.5 mM aurintricarboxylic acid in hybridization mix. The riboprobe was synthesized from eight approximately 1-kb templates using PCR primer sets with SP6 promoters amplified from B670 cDNA. Riboprobes synthesized from mac251 sequences did not detect SIV RNA^+^ cells in the brains of these animals (data not shown). After extensive washes and RNase treatment, tissue sections were dehydrated, coated in Kodak NTB emulsion, exposed at 4°C for 7 to 14 days, and developed and fixed per the manufacturer’s instructions. The fixed tissue sections were stained with hematoxylin, dehydrated, and mounted with Permount. Photographic images were taken with a digital camera, and the section areas were obtained by scanning slides with an Aperio CS2 scanner (Leica) (70). Densities of RNA^+^ cells per gram of tissue were calculated from the area of tissue examined, the 8-micron thickness (which determines the volume), and an assumed density of tissue (1.03 to 1.06 g/cm^3^, depending on the tissue).

### *In vitro* infection of peripheral blood mononuclear cells (PBMCs).

PBMCs from uninfected pig-tailed macaques were isolated by Percoll density gradient and plated in 48-well plates in RPMI media supplemented with 2 μg/ml recombinant human IL-2 (Life Technologies, Inc.) and 2 μg/ml PHA-M (Life Technologies, Inc.) overnight as previously described (35). PBMCs were subjected to spinoculation for 2 h (71) with 100-μl supernatants of SIV RNA-positive wells from brain MΦ-QVOAs. Media were replaced and supplemented with 2 μg/ml IL-2 (Life Technologies, Inc.). Supernatants were collected at days 5, 7, 10, and 13 postspinoculation. RNA was isolated from pooled samples (5 and 7 days and 10 and 13 days postspinoculation), and SIV RNA was quantitated by qRT-PCR.

### Mathematical model of biphasic viral decay.

The viral decay in plasma and CSF was modeled using a two-exponential form as described previously (22, 72). Briefly, the decay in the first 3 months posttreatment is biphasic—representing a brief, fast, first-order decline in viral load, followed by a longer, slower, first-order decline. For each cohort, the geometric means of measurements for each time point were used to generate a four-parameter fit of the form as follows: *V*(*t*) *= V*0 (*A e*^−µ*1t*^ + *B e*^−µ*2t*^), where *V*0 is the pretreatment viral load. The parameter fits were obtained using the trust region reflective algorithm (*lsqnonlin*) in MatLab (MathWorks, Inc., Natick, MA). We evaluated the half-lives of the two decay phases using the terms μ_1_ and μ_2_ as follows: *t*_1/2_ = *ln*(2)/μ_*i*_. The differences in parameters A and B between cohorts are less significant to the system than the decay rates (µ).

### Statistics.

Infected cell frequencies in limiting dilution assays were calculated using the IUPMStats v1.0 infection frequency calculator (http://silicianolab.johnshopkins.edu) (54). Statistics analyses were performed using Prism software (GraphPad Software, Inc., La Jolla, CA).
